# Eye Strain and the Rest Cure

**Published:** 1899-07-08

**Authors:** 


### EYE STRAIN AND THE REST CURE.

Dr. George Gould, of Philadelphia, draws attention to a certain fallacy in regard to the use of the "rest cure," and suggests that the benefit which is obtained thereby is really due in some cases to the coincident rest which is secured to the eyes by the form of treatment undergone. Thousands of patients, he says, are being treated, sometimes for organic and sometimes for functional disorders, whose ailments really spring from ocular defects. This, we think, is generally allowed, but he scores a point when he says that to mydriaticise a pair of eyes for a month or two would often do more good, would certainly be more logical, and would be an infinitely better means of differential diagnosis in obscure nerve trouble and functional nutritional disease, than to put the patient to bed for the same time. On the other hand we can imagine a person who is condemned to a couple of months of practical blindness saying, "then I might as well go to bed." Perhaps the real moral of Dr. Gould's suggestion is that "nerve cases" should have their eyes, as well as all other seats of possible eccentric irritation investigated and their defects relieved, before they are condemned to a "rest cure," a treatment which, after all, is not without its disadvantages, not the least of which is that, like tippling, some women fly to it again and again for small excuse.

